# Inflammation-Induced Tumorigenesis in Mouse Colon Is Caspase-6 Independent

**DOI:** 10.1371/journal.pone.0114270

**Published:** 2014-12-03

**Authors:** Bénédicte Foveau, Lauren Van Der Kraak, Nicole Beauchemin, Steffen Albrecht, Andréa C. LeBlanc

**Affiliations:** 1 Department of Neurology and Neurosurgery, McGill University, Montreal, QC, Canada; 2 Lady Davis Institute for Medical Research, Sir Mortimer B. Davis Jewish General Hospital, Montreal, QC, Canada; 3 Goodman Cancer Research Centre and Departments of Biochemistry, Medicine and Oncology, McGill University, Montreal, QC, Canada; 4 Department of Pathology, McGill University, Montreal, QC, Canada; UMR INSERM U866, France

## Abstract

Caspases play an important role in maintaining tissue homeostasis. Active Caspase-6 (Casp6) is considered a novel therapeutic target against Alzheimer disease (AD) since it is present in AD pathological brain lesions, associated with age-dependent cognitive decline, and causes age-dependent cognitive impairment in the mouse brain. However, active Casp6 is highly expressed and activated in normal human colon epithelial cells raising concerns that inhibiting Casp6 in AD may promote colon carcinogenesis. Furthermore, others have reported rare mutations of Casp6 in human colorectal cancers and an effect of Casp6 on apoptosis and metastasis of colon cancer cell lines. Here, we investigated the role of Casp6 in inflammation-associated azoxymethane/dextran sulfate sodium (AOM/DSS) colon cancer in Casp6-overexpressing and -deficient mice. In wild-type mice, AOM/DSS-induced tumors had significantly higher Casp6 mRNA, protein and activity levels compared to normal adjacent colon tissues. Increased human Casp6 or absence of Casp6 expression in mice colon epithelial cells did not change colonic tumor multiplicity, burden or distribution. Nevertheless, the incidence of hyperplasia was slightly reduced in human Casp6-overexpressing colons and increased in Casp6 null colons. Overexpression of Casp6 did not affect the grade of the tumors while all tumors in heterozygous or homozygous Casp6 null colons were high grade compared to only 50% high grade in wild-type mice. Casp6 levels did not alter cellular proliferation and apoptosis. These results suggest that Casp6 is unlikely to be involved in colitis-associated tumors.

## Introduction

Caspases are a family of intracellular cysteine proteases that play an important role in tissue homeostasis through modulation of inflammation and apoptosis. Dysregulation of these proteases result in inflammatory disorders, neurodegenerative diseases, and cancer [1].

Caspase-6 (Casp6) is classified as an effector caspase as it shares the short pro-domain feature common to other effector caspases, Casp3, and Casp7 [1]. However, Casp6 differs from Casp3 and Casp7 with respect to substrate specificity [2], its ability to self-activate intramolecularly [3] and its ability to be active in cells without causing apoptotic cell death [4], [5]. However, the exact role of Casp6 is still under investigation. Casp6 null mice have revealed a role for Casp6 in B cell activation and differentiation [6]. Neuronal Casp6 is implicated in axonal degeneration [7], [8], [9], [10], [11] and cleaves a number of neurocytoskeletal proteins [12]. Casp6 is also highly activated in neuritic plaques, neuropil threads, and neurofibrillary tangle lesions present in sporadic and familial forms of Alzheimer Disease (AD) [13], [14]. Higher levels of Casp6 activity in aged human brains are associated with lower cognitive performance and Casp6 expression in the hippocampus of transgenic mice can singly cause age-dependent spatial and episodic memory deficits [14], [15], [16], [17]. Furthermore, Casp6 has been implicated in both Huntington and Parkinson's disease [18], [19]. Together, these findings suggest that inhibition of Casp6 might be an efficient therapeutic approach against neurodegenerative diseases including cognitive decline in aged and AD individuals.

Casp6 is expressed almost ubiquitously in human tissues, although levels are decreased in aging tissues [20]. However, proCasp6 protein levels are higher in both fetal and adult colon compared to other human tissues. Strong active Casp6 was also detected in epithelial cells that slough off the colon epithelial lining. Previously, Casp6 activation has been implicated in intestinal epithelial cell anoikis, a programmed cell death caused by cellular detachment from the substratum [21], [22], [23]. Furthermore, proCasp6 protein levels are increased in human colon tumor tissues compared to adjacent normal tissues [20], 90% of colorectal cancers display moderate to strong Casp6 immunoreactivity ([24] and The Human Protein Atlas http://www.proteinatlas.org/ENSG00000138794/cancer/tissue). Rare mutations of the *CASP6* gene have been observed in human colon cancers [24]. Therefore, it is important to assess the role of Casp6 in colon carcinogenesis prior to pursuing the potential therapeutic inhibition of Casp6 in AD.

Other caspases previously have been implicated in colon carcinogenesis. In a mouse model of colitis-associated colorectal cancer induced by azoxymethane (AOM) and dextran sodium sulfate (DSS) treatment [25], Casp1-deficient mice showed enhanced inflammation-independent colon cancer formation due to dysregulation of colonic epithelial cell proliferation and apoptosis [26]. The fact that Casp1 can activate Casp6 [27], [28] raised the question of whether Casp6 is responsible for preventing Casp1-mediated colon carcinogenesis in this model. In this study, we directly investigated the role of Casp6 in AOM/DSS-induced colon carcinogenesis.

We found that Casp6 expression and activity were increased in colon tumors of Wild-Type (WT) mice. However, Casp6 overexpression or Casp6 deficiency did not influence the formation and the size of tumors induced by AOM/DSS treatment. Casp6 did not modulate cellular proliferation or apoptosis in AOM/DSS-induced tumors. These data show that Casp6 is unlikely to be involved in inflammation-induced colon carcinogenesis.

## Materials and Methods

### Mice

All animal procedures followed the Canadian Council on Animal Care guidelines and were approved by the McGill Animal Care Committee. A transgenic mouse model expressing a Cre-dependent self-activating human form of Casp6 (Knock-In mice, KI) was generated previously [16]. Expression of active Casp6 in the CAG promoter-Casp6^KI^ mouse model is transcriptionally silenced by an upstream loxP-flanked stop cassette. Once these mice are crossed with Villin-Cre (Cre) mice [29], obtained from Dr Nicole Beauchemin, the Cre recombinase allows expression of Casp6 specifically within the epithelial cells lining the colon by excising the stop sequence. The KI/Cre mice are a C57Bl6N background. Wild-Type (WT) and Casp6 Knock-Out (KO) mice were obtained from Jackson Laboratories (Bar Harbor, ME, USA) and are on a C57Bl6J background. All mice were bred in the pathogen-free Goodman Cancer Research Centre Mouse Transgenic Facility of McGill University.

### Murine inflammatory carcinogenesis protocol

**Figure 1:**
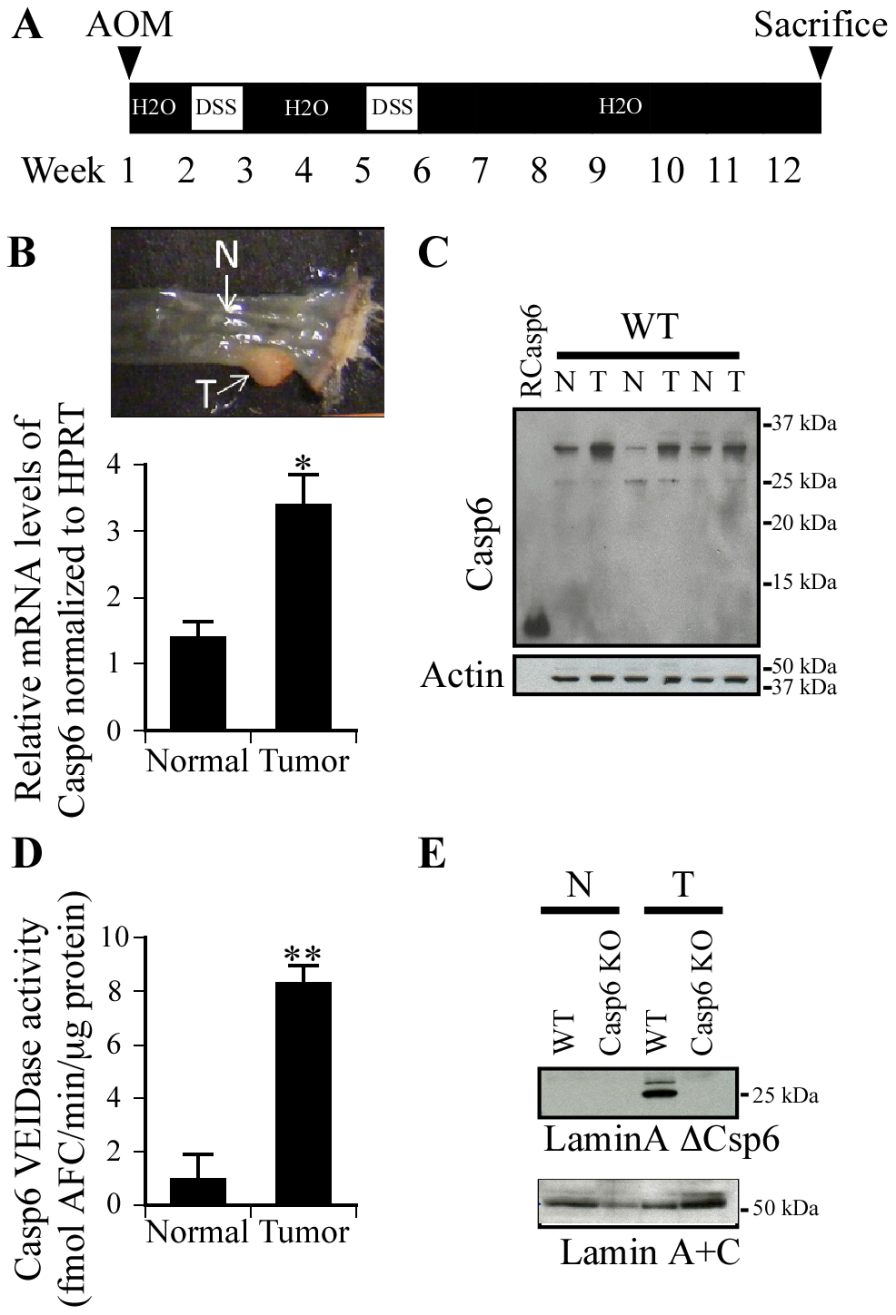
Casp6 levels are increased in AOM/DSS-induced colon tumors. (A) Schematic overview of the inflammation-induced cancer model. Age and sex-matched mice were injected with Azoxymethane (AOM) intraperitoneally at a dose of 6 mg/kg body weight. After one week, mice were treated with 2% DSS in the drinking water for 5 days, then followed by 16 days of regular water. This cycle was repeated once. Mice were sacrificed wither 12 or 20 weeks post AOM injection. (B) Casp6 mRNA was assessed by qRT-PCR on isolated RNA from tumors (T) and adjacent normal appearing tissues (N). HPRT was used as a loading control (n = 5). Statistical significance was assessed with a paired t-test. * p<0.05. (C) Casp6 protein expression in colon tissue extracts of WT mice was measured by western blot analysis with actin as a loading control (n = 3). (D) Specific VEIDase Casp6 activity in extracts from WT colons (n = 3). Statistical significance was assessed with a paired t-test. ** p<0.01. (E) Cleavage of lamin A by activated Casp6 was also tested using anti-lamin AΔCasp6 antibody and anti-lamin A+C used as equal loading control in tumors and normal appearing tissue from WT and KO Casp6 mice.

Mice were kept on wood chip bedding and given food (Charles River Chow 5075) and water ad libitum. Sex- and age-matched (10–11 weeks at the start of the experiment) co-housed mice were injected with a single intraperitoneal 6 mg/kg body weight of azoxymethane (AOM) (Sigma-Aldrich, St Louis, MO, USA) intraperitoneally. One week later, mice were treated with 2% dextran sulfate sodium (DSS Salt Reagent Grade MW 36,000–50,000, MP Biomedicals, Solon, OH, USA) in the drinking water for 5 days, followed by 16 days of regular water (**Fig. 1A**). This DSS cycle was repeated once. Animals showing signs of discomfort, such as weight loss, rectal bleed and prolapses, were sacrificed. All efforts were made to minimize suffering. All animals were euthanized with carbon dioxide at the same time. Colons were removed immediately, rinsed with PBS to remove fecal matter, sliced open longitudinally and analysed for the presence and surface area of tumors and hyperplasias. Colons were assessed in a blinded fashion under a stereo-dissecting microscope as previously described [30]. Tumors were measured using a clear transparency of 1 mm^2^ graph paper and the total surface area determined based on the total number of squares overlaying the tumor, as described [31].

### Histological analyses

After counting the number of tumors and hyperplasias, mouse colons were fixed with 10% neutral-buffered formalin for 24 h at room temperature (Fisher Scientific, Kalamazoo, MI, USA) and then embedded in the Swiss-roll orientation in paraffin [32]. Tissues were sectioned at 5 µm thickness and stained with eosin and hematoxylin (H&E). Tumor grading according to recommendations of Boivin et al. [33] and Washington et al. [34] was performed by Dr Steffen Albrecht, a pathologist. The grade of dysplasia was assessed on the most dysplastic area of each tumor.

### Total RNA extraction and Real-time Reverse-Transcriptase Polymerase Chain Reaction

Tumors and adjacent normal appearing colon tissues were dissected from experimental mice and total RNA was isolated using Trizol (Invitrogen, Carlsbad, CA, USA) according to the manufacturer's protocol. cDNA was prepared using avian myeloblastosis reverse transcriptase (AMV-RT) (Roche, Mannheim, Germany). Real-time PCR analysis was conducted using SYBR Green Taq Mastermix (Quanta BioSciences, Gaithersburg, MD, USA) on an Applied Biosystems 7500 Fast Real-Time PCR system machine. Results were normalized to Hypoxanthine-guanine phosphoribosyltransferase (*HPRT*) cDNA levels. Mouse Casp6 primers were purchased from Origene (Rockville, MD, USA) (MK204748). Human *CASP6* primers designed in house were as follows: sense 5′-GCATAAATGTGATTGCCTTCGC-3′; antisense 5′-AGATCTAGGATTTGAAGTGAAATGC-3′. The sequences for the *HPRT* primers [35] were: sense 5′-GTAATGATCAGTCAACGGGGGAC-3′; antisense 5′-CCAGCAAGCTTGCAACC TTAACCA-3′. Results were expressed as fold-induction values normalized to the *HPRT* control, using the Pfaffl's method [36].

### Caspase activity measurement and western blotting

Proteins from normal and tumor colon tissue were extracted in Cell Lysis Buffer (HEPES 50 mM, CHAPS 0.1%, EDTA 0.1 mM). The protein concentration was determined using a Bradford Assay (BioRad, Hercules, CA, USA). Casp6 activity was detected using the Ac-VEID-AFC (Ac-Val-Glu-Ile-Asp-7-Amino-4-trifluoromethylcouramin) (Enzo LifeSciences, NY, USA) substrate as previously detailed [37]. Once the enzyme activity assay completed, the reaction mix was boiled in Laemmli buffer, and 20 to 100 µg of proteins were loaded on a 10% or 15% SDS-polyacrylamide gels and analyzed by western blotting with monoclonal anti-Casp6_271-285_ antibody 556581 recognizing the full length and the p10 subunit of Casp6 (1/250, BD Pharmingen, Mississauga, ON, Canada), polyclonal anti-Casp6 10630 raised against the PLDVVD C-terminal amino acids of the p20 subunit (1/5000, laboratory-made anti-p20Casp6 10630 neoepitope antibody), polyclonal anti-lamin AΔCasp6 CS2035 raised against amino-terminal residues surrounding Asp230 in human lamin A (1/1000, Cell Signaling, Whitby, ON, Canada), monoclonal anti-lamin A+C_464-572_ Ab4789 (1/50, Abcam, Toronto, ON, Canada) and monoclonal anti-β-actin A5441 raised against the 16 N-terminal amino acids(1/5000, Sigma-Aldrich, St Louis, MO, USA). Immunoreactivity was revealed with horseradish peroxidase (HRP) or alkaline phosphatase (AP) conjugated secondary anti-mouse or anti-rabbit antibodies (1∶5,000, Jackson ImmunoResearch Laboratories, West Grove, PA, USA or GE Healthcare Bio-Sciences, Piscataway, NJ, USA) and ECL (Enhanced ChemiLuminescence) or NBT/BCIP (p-nitroblue tetrazodium chloride/5-bromo-4-chloro-3-indolyl phosphate) (GE Healthcare, EMD Millipore, Billerica, MA, USA, or Promega, Madison, WI, USA).

### Immunohistochemistry

Tissue sections were deparaffinized, rehydrated, and treated with Tris-EDTA antigen retrieval buffer (pH 9) for 20 min at 97 °C in the Pascal Dako Cytomation apparatus. For immunostaining, the Dako Autostainer Plus automated slide processor and the EnVision Flex system (Dako, Burlington, ON, Canada) were used as previously described [16]. Tissue sections were incubated with rabbit polyclonal anti-Ki67_1200-1300_ Ab15580 (1/2000, Abcam, Toronto, ON, Canada), polyclonal anti-Casp6 10630 raised against the PLDVVD C-terminal amino acids of the p20 subunit (1/3500, laboratory-made anti-p20Casp6 10630 neoepitope antibody) or rabit poluyclonal anti-cleaved Casp3 CS9661 raised against amino-terminal residues adjacent to (Asp175) in human caspase-3 (1/100, Cell Signaling, Danvers, MA, USA). Immunoreactivity was revealed with rabbit-HRP secondary antibody and diaminobenzidine (DAB) (Dako, Burlington, ON, Canada). Slides were counterstained with hematoxylin, dehydrated, mounted in Permount mounting medium (Fisher Scientific, Ottawa, ON, Canada) and scanned with the Mirax Scan (Zeiss, Don Mills, ON, Canada). Ki67- and Casp3-positive cells were counted in at least 3 high power fields (20X) in colon tumors using Aperio Imagescope software 11.1 for each colon. The results were expressed as staining-positive cells/mm^2^.

### Statistical analysis

The statistical significance of differences between experimental groups was analyzed using InStat3 software and Graphpad Prism software (GraphPad Software, La Jolla, CA, USA) as indicated in the figure legends. For all statistics, a p-value less than 0.05 is represented by *, a p value less than 0.01 is represented by **, and a p-value less than 0.001 is represented by ***. Chi-square test was used to analyze the incidence of tumors and grade of dysplasia.

## Results

### Casp6 expression and activity are increased in AOM/DSS tumors

To determine if Casp6 may be involved in colon carcinogenesis, we treated mice with AOM/DSS. Wild-type male and female mice were injected with AOM (6 mg/kg) and treated with two cycles of 2% DSS (**Fig. 1A**). Casp6 gene expression was analysed by quantitative Real-Time Polymerase Chain Reactions (qRT-PCR) in tumors and adjacent normal colon tissue (**Fig. 1B**). Casp6 mRNA levels increased 2 fold (p<0.05) in colon tumors compared to adjacent normal tissues (**Fig. 1B**). ProCasp6 protein levels also increased in AOM/DSS tumors, but the p10 and p20 active subunits of Casp6 were not detected by western blotting, consistent with the rapid degradation of active caspase subunits by the proteasome [38] (**Fig. 1C**). Nevertheless, Casp6 activity, measured with the Ac-VEID-AFC fluorogenic substrate increased 8 fold (p<0.01) in tumors compared to normal adjacent tissue (**Fig. 1D**). Casp6 activity was confirmed in tumors but not in normal tissue by the detection of laminΔA cleaved by Casp6 (lamin AΔCasp6) since lamin A is the prototypical substrate of Casp6 [39](**Fig. 1E**). Together, these results indicate that Casp6 mRNA, protein and activity levels are increased in AOM/DSS-induced colon tumors.

### Casp6 expression in mice colons does not alter the susceptibility to AOM/DSS-induced carcinogenesis

**Figure 2:**
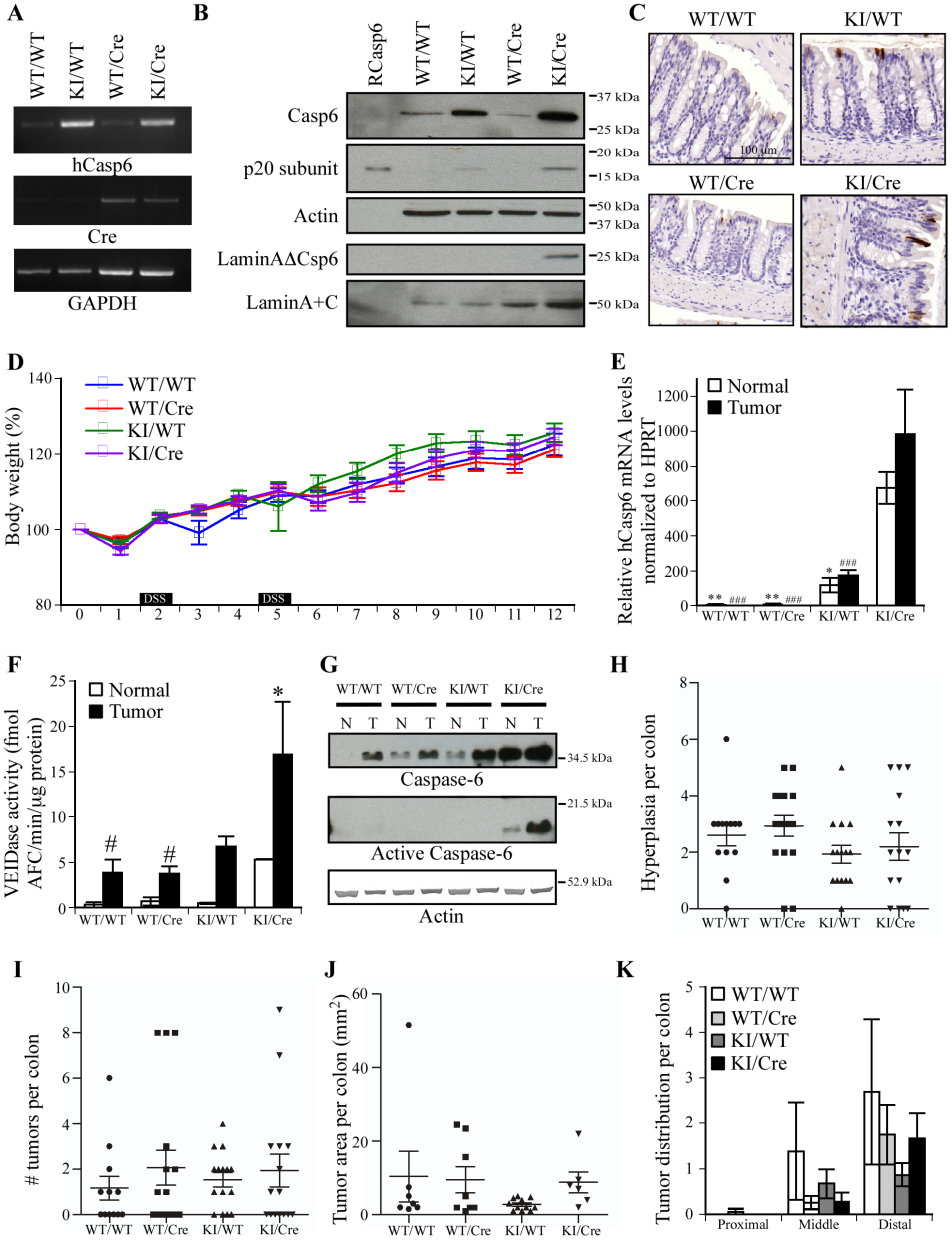
Susceptibility of Casp6 overexpressing mice to AOM-DSS treatment. (**A**) RT-PCR of human Casp6 and Cre recombinase in colons of KI and Cre mice. (B) Western blot of human Casp6 protein and active subunits in mice colon proteins. Cleavage of lamin A by activated Casp6 was also tested using anti-lamin AΔCasp6 antibody and anti-lamin A+C used as equal loading control. (C) Immunohistochemistry of WT/WT, KI/WT, WT/Cre and KI/Cre mice colons with anti-p20 active Casp6 neoepitope antisera. (D) Percent weight change measured weekly during AOM-DSS treatment. The black boxes represent the period of DSS treatment. (E) Human Casp6 mRNA levels were assessed by qRT-PCR in tumors and adjacent normal appearing tissue from WT/WT, WT/Cre, KI/WT and KI/Cre mice colons. HPRT was used as a loading control (n = 3). One-way ANOVA followed by Tukey-Kramer post hoc analysis was performed to determine statistical significance. * p<0.05, ** p<0.01 comparing with Normal KI/Cre and ### p<0.001 comparing with Tumor KI/Cre. (F) Specific VEIDase Casp6 activity for extracts from WT/WT, WT/Cre, KI/WT and KI/Cre colons (n = 3). One-way ANOVA followed by Tukey-Kramer post hoc analysis was performed to determine statistical significance. * p<0.05 compares normal versus tumor tissues and # p<0.05 compares with tumors from KI/Cre. (G) Western blot analyses for pro-Casp6 and active p20 subunit of Casp6 in colon protein extracts from WT/WT, WT/Cre, KI/WT and KI/Cre mice. (H) Number of hyperplasia per colon induced by AOM-DSS treatment 12 weeks after AOM injection. (I) Number of tumors per colon induced by AOM-DSS treatment 12 weeks after AOM injection. (J) Tumor load per mouse in mice after AOM/DSS treatment. (K) Number of tumors per mouse located in proximal, middle or distal part of colons. (H-K) Statistics were performed using one way ANOVA followed by Tukey-Kramer post hoc analysis.

To determine the impact of Casp6 in colon, we generated mice conditionally expressing a self-activated form of Casp6 (p20p10Casp6) under the regulation of the Villin promoter in the colon (Knock-In/Villin-Cre; KI/Cre) based on a previously established transgenic Casp6 mouse [16]. These KI/Cre mice over-express Casp6 mRNA (**Fig. 2A**), pro-Casp6 and its p20 active subunit (**Fig. 2B**) in the colon and immunohistochemistry revealed expression of active Casp6 in the epithelial cell lining of the colon [29] (**Fig. 2C**). The KI/WT also showed leaky expression of the human Casp6 transgene mRNA (**Fig. 2A**), and protein (**Fig. 2B&C**), but levels of p20Casp6 active subunit were not detectable in western blots (**Fig. 2B**). Casp6 over-expressing KI/Cre mice did not develop any symptoms or macroscopically detectable tumors in their colon for up to 18 months of age (n = 3 examined for each of the four genotypes). The KI/Cre and control wild-type (WT/WT), Villin-Cre (WT/Cre), and transgenic Casp6 KI without Cre expression (KI/WT) littermates were subjected to AOM/DSS-induced carcinogenesis. No apparent differences were noted with respect to weight loss or gain in the different genotypes (**Fig. 2D**). Almost all mice survived after the DSS treatments indicating no difference in sensitivity to DSS exposure for the different genotypes. Irrespective of genotype, all mice developed rectal bleeds and prolapses consistent with colorectal cancer and were sacrificed 12 weeks after the initial AOM injection. Over-expression of human p20p10Casp6 in KI/Cre mice colons was confirmed by qRT-PCR (**Fig. 2E**). As expected, VEIDase Casp6 activity levels (**Fig. 2F**) and pro-Casp6 and active Casp6 p20 subunits (**Fig. 2G**) were significantly higher in KI/Cre than in the control mice normal and tumor tissues. Interestingly, despite higher levels of Casp6 expression in the KI/WT, these mice did not show increased Casp6 activity and protein levels following the AOM/DSS treatment compared with the other two control groups (**Fig. 2F&G**). The impact of Casp6 overexpression on colitis-associated tumor development was measured by scoring two types of macroscopic lesions on colons: hyperplasic lesions represented by a thickening patch in the colon, often in the shape of a donut, and tumors identified as protruding masses with thick and opaque interior [30]. Hyperplasias were found in 86% to 90% of the three control groups and in 75% of the KI/Cre group (**Table 1A**). Tumors were present in approximately 50% of WT/WT, WT/Cre and KI/Cre mice but were increased to 80% of the KI/WT mice (**Table 1A**). These differences did not reach statistical significance, however. Similarly, no statistically significant difference was observed for the average hyperplasic and tumor numbers per colon between the 4 groups of mice tested (**Fig. 2H&I**). The tumor surface area per mouse (**Fig. 2J**) and the distribution of tumors along the colon (**Fig. 2K**) did not differ significantly between the different genotypes. The lesions observed were mainly distributed in the distal and middle portions of the colon as expected for the AOM/DSS model (**Fig. 2K**). Therefore these results indicate that Casp6 overexpression in the colon has no impact on tumor development in the AOM/DSS-induced colon carcinogenesis.

**Table 1:** Hyperplasia and Tumor (macroscopic) incidence (%) in KI/Cre (A) and KO (B) mice treated with AOM/DSS.

	Genotype	Number of mice examined	Incidence (%)	
			Hyperplasia	Tumors
A	**WT/WT**	15	86.67	53.3
	**WT/Cre**	16	87.5	50
	**KI/WT**	14	93.3	80
	**KI/Cre**	15	75	53.3
B	**WT/WT**	15	86.7	40
	**WT/KO**	17	82.3	64.7
	**KO/KO**	9	100	66.7

Data on the hyperplasia and tumors incidence were analyzed by X^2^ test. Differences were considered significant if the probability of the difference by chance was less than 5 in 100 (P<0.05).

### The absence of Casp6 expression in mice does not alter the susceptibility to AOM/DSS induced carcinogenesis

**Figure 3:**
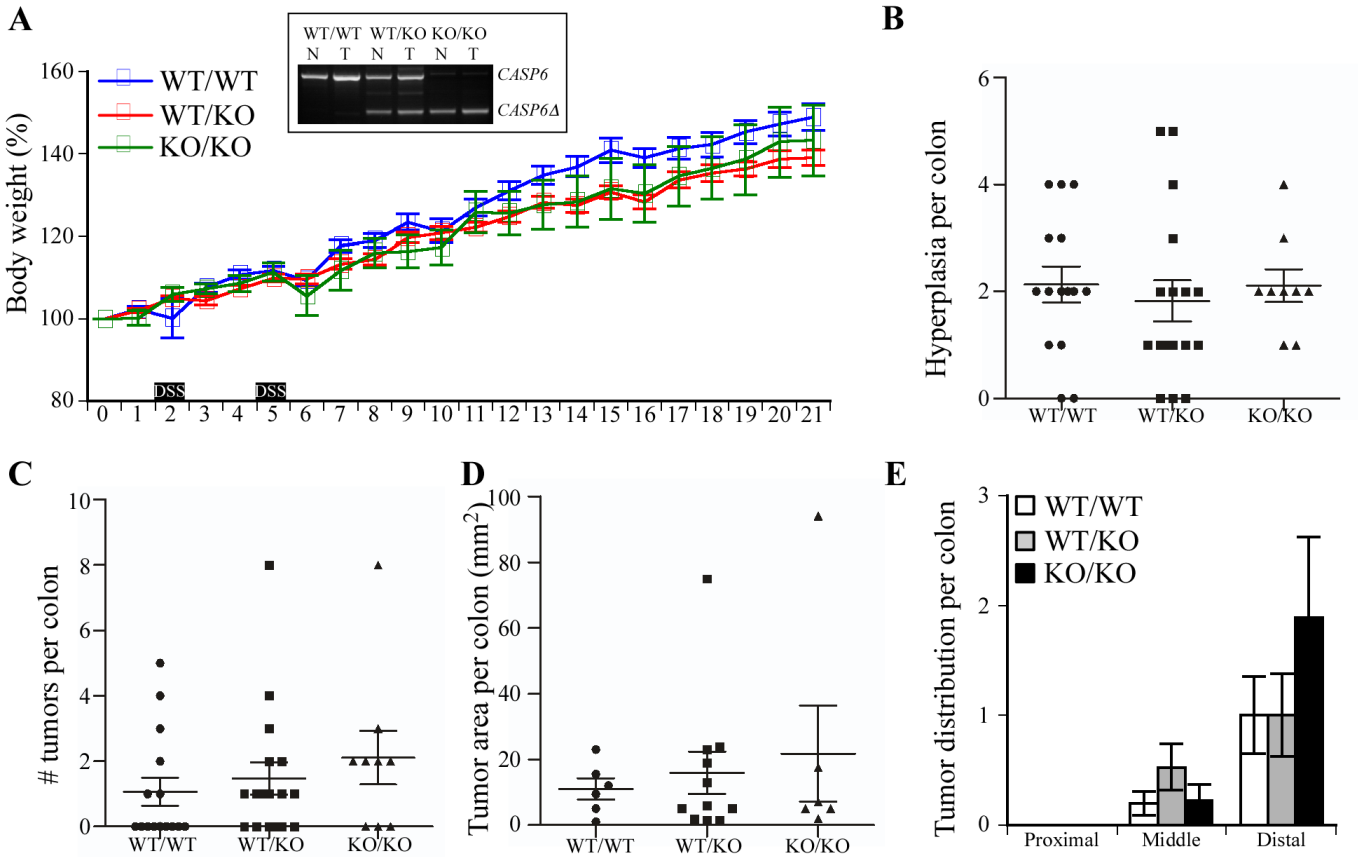
Susceptibility of Casp6 deficient mice to AOM/DSS treatment. (A) Percent weight change measured weekly during AOM-DSS treatment. The black boxes represent the period for DSS treatment. Inset: Casp6 mRNA was assessed by RT-PCR with primers amplifying full length Casp6 mRNA in WT and truncated Casp6 mRNA in the KO. (B) Number of hyperplasia per colon induced by AOM-DSS treatment 20 weeks post AOM injection. (C) Number of tumors per colon induced by AOM-DSS treatment 20 weeks after AOM injection. (D) Tumor load per mouse in mice after AOM/DSS treatment. (E) Number of tumors per mouse located in proximal, middle or distal part of colons. (B-E) Statistics were performed using one way ANOVA followed by Tukey-Kramer post hoc analysis.

Mice deficient in Casp6 (KO/KO) were submitted to the same AOM/DSS treatment to assess if the absence of Casp6 expression affected tumorigenesis. During the experiment, average body weight did not differ in KO/KO mice compared to WT/WT and WT/KO mice treated with AOM/DSS (**Fig. 3A**). Genotypes were verified by RT-PCR (**Fig. 3A** **inset**). At the onset of symptoms, twenty weeks post-AOM injection, mice were sacrificed, and colons dissected. Macroscopically visible lesions counted under the stereomicroscope showed hyperplasia in 86.7% and 82.3% of the WT/WT and WT/KO colons, whereas hyperplasia were observed in 100% of the KO/KO mice (**Table 1B**). The incidence of colonic tumors increased by 25% in WT/KO and KO/KO mice compared to WT counterparts but this value did not reach statistical significance in a Chi-square test (**Table 1B**). Following AOM/DSS treatment, WT/WT and KO/KO mice had nearly identical numbers of hyperplasia (around 2 per mouse) and tumors (1 to 2 per mouse) (**Fig. 3B & C**). No significant difference was observed in the tumor burden (**Fig. 3D**) or tumor distribution in proximal, distal and middle colon sections (**Fig. 3E**). These results suggest no significant effect of endogenous Casp6 in this model of inflammation-associated cancer development.

### Cellular proliferation is not altered by the expression of Casp6 in AOM-DSS-treated mice colons

**Figure 4:**
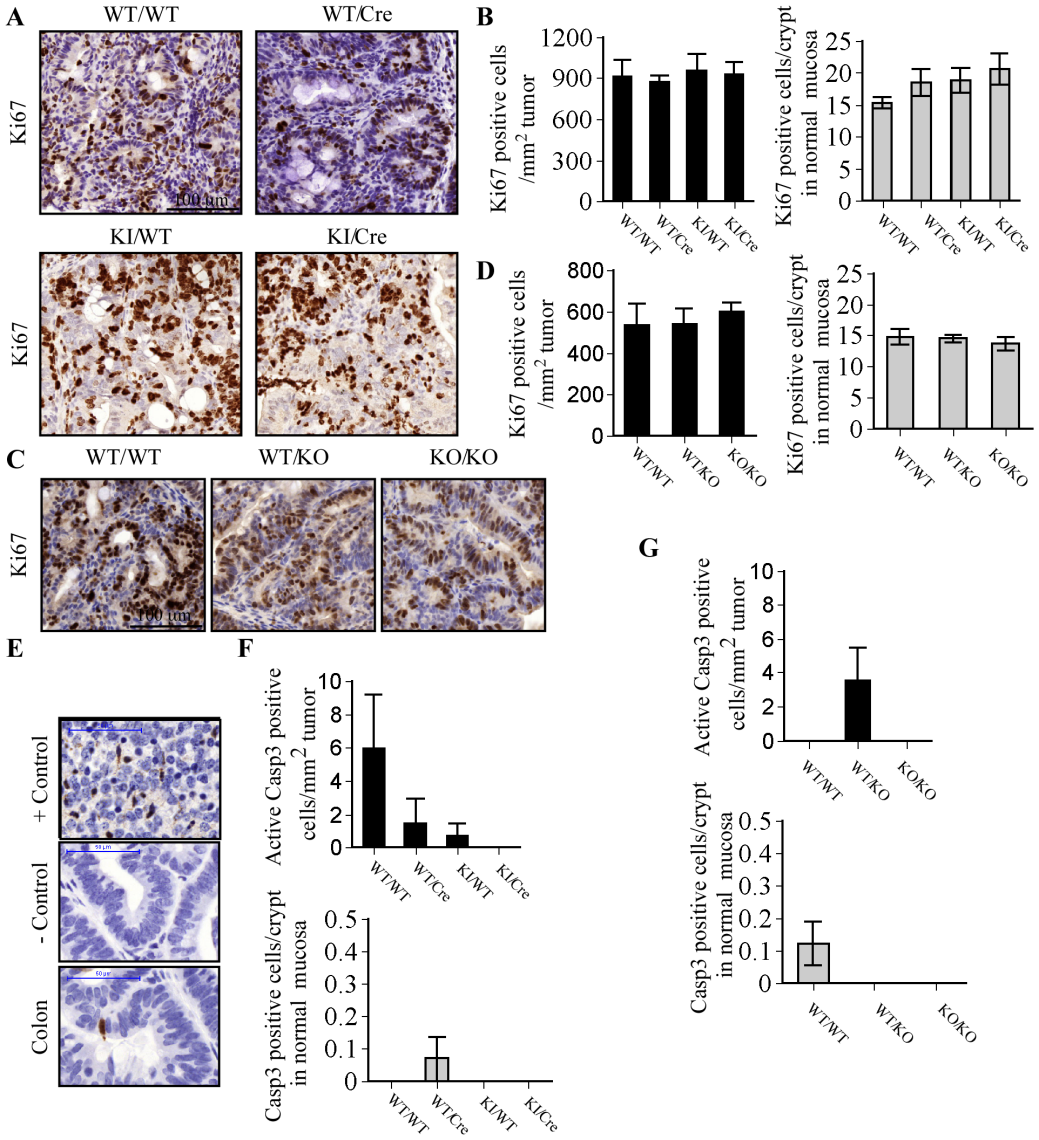
Tumor cell proliferation and apoptosis in Casp6 overexpressing and deficient mice. (A) Representative Ki67 immunohistochemistry in tumors in WT/WT, WT/Cre, KI/WT and KI/Cre mice given AOM/DSS (scale bar, 100 µm). (B) Number of Ki67 positive cells in colonic tumors from mice (N = 3). Data expressed as means +/− SEM. (C) Representative Ki67 immunohistochemistry in tumors from WT/WT, WT/KO, and KO/KO mice given AOM/DSS (scale bar, 100 µm). (D) Number of Ki67 positive cells in colonic tumors from KO mice (N = 3). Data expressed as means +/− SEM. (E) Representative Cleaved Casp3 immunohistochemistry in positive control (fetal ischemic brain), negative control (colon without Primary Antibody) and a colon from AOM/DSS treated mouse (scale bar, 50 µm). Number of cleaved Casp3 positive cells in colonic tumors from mice (N = 3). Data expressed as means +/− SEM. (F) Number of Cleaved Casp3 positive cells in colonic tumors from KO mice (N = 3). Data expressed as means +/− SEM.

Histological analysis showed no histopathological difference in tumors from WT/WT and WT/Cre control mice and KI/Cre Casp6 overexpressing mice. However, only high-grade dysplasia (HGD) were found in KI/WT mice (**Table 2A**). Chi-square analysis did not reveal a significant difference between the groups. The heterozygous and homozygous Casp6 KO mice tumors were 100% high grade tumors compared to 50% low grade and high grade in the WT/WT control mice (**Table 2B**). Irrespective of genotype, none of the lesions invaded the muscularis mucosa and thus did not develop into adenocarcinoma. Ki67 immunohistochemistry was performed to determine the impact of mice genotype on cellular proliferation in tumors (**Fig. 4A**). The number of Ki67 positive cells was similar in the tumors from WT/WT, WT/Cre, KI/WT and KI/Cre mice treated with AOM/DSS (**Fig. 4B**). Similarly, no significant difference was found in the number of Ki67 positive cells in WT/WT, WT/KO and KO/KO mice tumors (**Fig.4C&D**). To assess apoptosis, tumor sections were stained for active Casp3. While the positive ischemic brain control showed ample Casp3 immunopositive cells, few were observed in the mouse colonic tumors (**Fig. 4E**). Quantitative analyses did not reveal any significant differences between Casp3 positive cells in the colon tumors from different mice genotypes (**Fig. 4F & G**). These results suggest that Casp6 expression does not modulate cellular proliferation in the AOM/DSS-induced carcinogenesis model.

**Table 2:** Histological grades of AOM/DSS-induced colon tumors in KI/Cre (A) and KO (B) mice.

	Genotype	Number of mice examined	Number of Adenoma		
			Total	LGD	HGD
A	**WT/WT**	4	5	2: 40%	3: 60%
	**WT/Cre**	8	7	4: 57%	3: 43%
	**KI/WT**	5	5	0: 0%	5: 100%
	**KI/Cre**	14	11	5: 45%	6: 55%
B	**WT/WT**	6	3	1: 33%	2: 66%
	**WT/KO**	7	5	0: 0%	5: 100%
	**KO/KO**	6	4	0: 0%	4: 100%

Abbreviations: LGD, adenoma with low-grade dysplasia; HGD, adenoma with high-grade dysplasia.

## Discussion

In this paper, we evaluated the role of Casp6 in colon carcinogenesis because (1) Casp6 is observed to be highly expressed in human fetal and adult colons [20], (2) Casp6 is increased in human colon cancers compared to adjacent normal tissues [20], (3) rare mutations of Casp6 were observed in human colorectal cancers [24], and (4) Casp6 in implicated in the apoptosis of colon cancer cell lines [40], [41], [42], [43], [44]. The AOM/DSS colon carcinogenesis model was chosen for our study because Casp1 represses colon carcinogenesis in this model by increasing cell death and reducing cellular proliferation rather than acting through inflammation [26], [45] and Casp1 can activate Casp6 [27]. Consistent with increased Casp6 expression and activity in human colon cancers, the AOM/DSS-induced colon cancers in mice expressed higher levels of Casp6 mRNA, protein and activity compared to normal adjacent colon tissues. Similarly, the APC(Min/+) mutant model of mouse colon carcinogenesis has an increase of Casp6 mRNA in tumors (Geo profile GDS389 [46]). Therefore, these finding incited us to directly address the role of Casp6 in colon carcinogenesis by treating Casp6 overexpressing transgenic mice and Casp6 null mice with AOM/DSS. While we observed a trend towards an increase in the incidence of hyperplasias numbers, tumor numbers, and tumor grade in the absence of Casp6 gene expression, and a reduction of hyperplasias in the transgenic overexpressing Casp6 mice, the results did not reach statistical significance. Furthermore, we excluded any differences in the number of hyperplasias and tumors per colon and their distribution along the colon in Casp6 overexpressing transgenic mice and Casp6 null mice. No differences were observed in the proliferation or apoptosis of cells in the colonic tumors. We therefore conclude that the increased expression of Casp6 does not influence AOM/DSS-induced colon carcinogenesis and is not responsible for the Casp1-dependent resistance to colon carcinogenesis in this model.

Nevertheless, the increased Casp6 gene expression and activity in colon tumors is likely affecting colon physiology. Casp6 activation is a crucial signaling mechanism in apoptosis triggered by some chemotherapeutics agents such as scutellarin [41], resveratrol [40], [42] and aloe emodin [44]. In addition, p53-dependent Casp6 overexpression and activity participated in chemosensitization of cells to Adriamycin [47]. Possibly, Casp6 may constitute a defense mechanism to promote cell demise in specific situations.

The fact that Casp6 is not involved in inflammation-induced colon tumorigenesis does not rule out a possible involvement of Casp6 in metastasis. Several of the Casp6 substrates are cytoskeletal proteins including alpha-tubulin and lamin A, suggesting that Casp6 activity may affect cell shape and motility [39], [42], [48], [49]. Casp6 is implicated in anoikis, and resistance to anoikis is a critical mediator of metastasis in cancer [50] Unfortunately, the colorectal cancer AOM/DSS model is not metastatic [51], so the metastatic aspect of cancer could not be addressed in this paper.

In conclusion, Casp6 is strongly expressed and activated in tumors formed in a mouse model of colitis-associated tumorigenesis. However, AOM/DSS-induced colon carcinogenesis is not strongly Casp6-dependent. Our results suggest that inhibitors of Casp6 could be considered as novel therapeutic approaches for neurodegenerative diseases without a high risk of carcinogenic side-effects.
